# Inconsistency of Association between Coffee Consumption and Cognitive Function in Adults and Elderly in a Cross-Sectional Study (ELSA-Brasil)

**DOI:** 10.3390/nu7115487

**Published:** 2015-11-19

**Authors:** Larissa Fortunato Araújo, Luana Giatti, Rodrigo C. Padilha dos Reis, Alessandra C. Goulart, Maria Inês Schmidt, Bruce B. Duncan, Mohammad Arfan Ikram, Sandhi Maria Barreto

**Affiliations:** 1 Research Group on Epidemiology on Chronic and Occupational Diseases (GERMINAL), School of Medicine, Universidade Federal de Minas Gerais, Belo Horizonte, Minas Gerais 30130-100, Brazil; larissafortunatoaraujo@gmail.com (L.F.A.); luana.giatti@gmail.com (L.G.); rodrigocpdosreis@gmail.com (R.C.P.R.); 2 Center for Epidemiological and Clinical Research, Hospital Universitário, Universidade de São Paulo, São Paulo 05508-000, Brazil; agoulart@hu.usp.br; 3 Postgraduate Studies Program in Epidemiology, School of Medicine, Universidade Federal Rio Grande do Sul, Porto Alegre, Rio Grande do Sul 96203-900, Brazil; maria.schmidt@ufrgs.br (M.I.S.); bbduncan@ufrgs.br (B.B.D.); 4 Department of Epidemiology, Erasmus University Medical Center, Rotterdam 3000 CA, The Netherlands; m.a.ikram@erasmusmc.nl

**Keywords:** coffee consumption, diet bioactive compounds, cognitive function tasks

## Abstract

**Background:** Coffee is one of the most consumed beverages worldwide and the effect on cognition appears to be task specific and vary by age. **Method:** In cohort of 14,563 public service workers (35–74 years old) we assessed coffee consumption habits and examined cognitive function using standardized neuropsychological test battery. By linear regression and generalize linear regression with logarithmic link and gamma distribution we investigated the relation of coffee consumption (never/almost never, ≤1 cup/day, 2–3 cups/day, ≥3 cups/day) in the last 12 months to performance on specific domains of cognition for adults and elderly separately. **Results:** Among elderly, after adjustments, coffee consumption was associated only with an increase in the mean words remembered on learning, recall, and word recognition tests when comparing the 2–3 cups/day to never/almost never category (arithmetic mean ratio (AMR): 1.03; 95% Confidence Interval (CI): 1.00 to 1.07), and to an increase in the mean words pronounced in semantic verbal fluency test when comparing the ≥3 cups/day to never/almost never category (difference of the mean: 1.23; 95% CI: 0.16 to 2.29). However, coffee consumption was not associated with any cognitive function tests in adults and also was not associated with the phonemic verbal fluency test and trail-making test B in elderly. **Conclusions:** Results suggest that coffee consumption might be slightly beneficial to memory in elderly but lacks a dose response relationship. Longitudinal analyses are needed to investigate possible, even if subtle, positive effects of coffee drinking on specific cognitive domains in elderly.

## 1. Background

There is a general agreement on the existence of a normal cognitive decline from early to late adulthood and that disorders, such as Alzheimer’s disease (AD), are associated with an overall impairment of higher functions and cognitive faculties, one of which is a symptomatic loss of memory [1]. In recent years, based on a possible link between oxidative damage and cognitive decline, there has been a growing interest in studying bioactive diet compounds that could delay or prevent cell damage, provide symptomatic relief, and improve people’s quality of life [2].

Coffee is a very popular beverage consumed worldwide and Brazil ranks first among coffee producer countries and second among the coffee consumer countries [3]. It is a rich source of caffeine, which acts as a psychoactive stimulant and has been shown to improve heightened alertness, vigilance, attention, mood as well as complex, higher cognitive functions [4,5,6,7]. Although, results from previous studies of the effect of caffeine on cognition among the elderly have been inconsistent, indicating that caffeine may have either a facilitating or a detrimental effect on cognition and that the effect of caffeine may be task specific, such as memory, language, or executive function [8,9,10,11,12].

The most convincing of the epidemiologic studies in establishing an association between caffeine and AD reported that AD patients consumed markedly less caffeine during the 20 years preceding diagnosis of AD, compared with age-matched individuals without AD [13]. Still, whether and how coffee consumption is related to better cognition remains unclear. Moreover, studies on coffee and cognitive function in younger adults are very scarce. The present study aims to the association of coffee consumption in the last 12 months with specific domains of cognitive function among Brazilian adults and elderly.

## 2. Method

### 2.1. Setting and Study Population

The Longitudinal Study of Adult Health (ELSA-Brasil) was established in 2008 as a longitudinal study to examine development and progression of clinical and subclinical chronic diseases, particularly cardiovascular diseases and diabetes among 15,105 civil servants from universities and research institutes in six Brazilian cities [14,15]. This cross-sectional study used data regarding participants who undertook cognitive tests in the baseline stage, those that reported previous diagnosis of stroke (*n* = 184) and/or were using neuroleptics, anticonvulsants, anticholinesterase or antiparkinsonian drugs were excluded from the present analysis (*n* = 330). From 14,591 study participants with data on cognitive function, 14,563 had information about coffee consumption. Only 316 persons indicated they drank coffee without caffeine and were not excluded from the analysis since coffee has antioxidant compounds that can potentially influence cognition as well. Not all persons underwent all cognitive tests, resulting in slightly different totals for learning, recall and word recognition tests (*n* = 14,451), semantic and phonemic verbal fluency tests (*n* = 14,532 and *n* = 14,558, respectively), and trail-making B test (*n* = 13,139) composing the total in each analysis. The study was approved by the Committee of Ethics in Research (approval No. 189/2006). All participants gave their informed consent to participate.

### 2.2. Measurement of Coffee Consumption

Dietary data were collected using a validated semi quantitative food frequency questionnaire (FFQ) that indicated all foods and drinks in the last 12 months. Reproducibility and relative validity of the FFQ compared to three food records completed by a sub-set of participants and assessed through the intraclass correlation coefficient (ICC) showed moderate to good reproducibility ranging from 0.55 to 0.83 and relative validity ranging from 0.20 to 0.72, depending on the nutrient [16]. Participants were further asked to specify the type of coffee normally consumed (filter, instant, espresso, moka pot), whether this coffee contained caffeine (caffeinated or decaffeinated), whether additional items were typically added to the coffee (sugar, artificial sweetener), and the typical quantity of coffee consumed on each occasion in relation to a reference cup size of 50 mL. Answer choices were provided as ordinal categories: “More than 3 times per day”, “2–3 times per day”, “once a day”, “5–6 times per week”, “2–4 per week”, “once a week”, 1–3 times a month” and “never or almost never”. The dietary coffee consumption was converted into three subgroups: “Never/almost never”, “≤1cup/day”, “2–3 cups/day”, “≥3 cups/day”.

### 2.3. Cognitive Test Battery

The response variables were the final scores obtained in selected the following cognitive function tests of the Consortium to Establish a Registry for Alzheimer’s Disease (CERAD) [17], validated for the elderly Brazilian population. The **learning recall and word recognition tests** were used to evaluate verbal learning, retrieval from verbal memory, and recognition of verbal memory. The score corresponds to the sum of the correct words (range: 0–50). The **semantic (animal category) and phonemic (letter F) verbal fluency tests** were used to evaluate efficiency of searching in long-term memory and language and the score corresponds to the total number of correct animal names and words beginning with the letter “F” given by the participant. The **trail-making test B** was used to evaluate executive function, as it is related to attention, concentration, and psychomotor speed [18]. Trail-making test A was used to train the participants. The score corresponded to the time (in seconds) taken to complete trail-making test B with limit of 300 seconds.

The higher the scores on the learning, recall, and word recognition tests and on the semantic and phonemic verbal fluency tests the better cognitive function in those domains. On the other hand, the longer the time taken to complete the trail-making test B the worse the participant’s performance. The reliability of these tests varied from moderate, for the learning and word recall test (*Kappa* = 0.56; 0.33–0.79), to very good, for the trail making test B (*Kappa* = 0.91; 0.87–0.95) [19].

### 2.4. Measurement of Confounders

All the confounders included in this analysis were self-reported through standardized questionnaires or obtained through clinical procedures or laboratory exams measurements [14,20]*.* To control for confounding, we selected established risk factors for cognitive performance that were also known to be associated with coffee consumption based on prior studies and theoretical considerations. They were: *age* in years; *sex* (male and female); c*urrent educational attainment* was assessed as the highest qualification attained (graduate school or more, complete high school, complete elementary school and incomplete elementary school); *alcohol consumption* (never or former user, moderate, heavy); *smoking status* (current smoker how had smoked at least 100 cigarettes (five packs of cigarettes) throughout life and still being a smoker, former, and never); *diabetes status* (fasting glucose ≥126 mg/dl, and/or glycated hemoglobin >6.5 mL/dl, and/or 2 h post glucose ≥200 mg/dl, and/or self-report of a physician diagnose, and/or use of insulin or oral antidiabetics medication); *hypertension* *status* (defined as systolic pressure ≥140 mmHg, and/or diastolic ≥90 mmHg, and/or confirmed drug treatment for hypertension); *coronary heart disease status* (report of myocardial revascularization and/or myocardial infarction); and *Low-density lipoprotein cholesterol level (g/mL) (LDL-Cholesterol)*.

### 2.5. Data Analysis

We performed stratified analysis to explore if the relation between coffee consumption and the cognitive domains was different between adults (35–64 years old) and the elderly (65–74 years old). The characteristics of the study population are presented using unadjusted means (Stardant Deviation (SD)), medians (1° and 4° quartiles) and frequencies by category of coffee consumption, and were performed Chi-square for frequencies, analysis of variance (ANOVA) for normal distribution and KrusKal-Wallis for non-normal distribution.

Two strategies of analysis were employed to investigate the association between the amount of coffee intake and the performance on each of the outcome variables. We used multiple linear regressions for the semantic and phonemic verbal fluency tests because their scores presented normal distributions. We used a generalized linear model (GLM) with logarithmic link and gamma distribution to measure the differences between categories of explanatory variables for the outcomes learning, recall and word recognition tests and the number of seconds taken to complete the trail-making B test because of the skewed distribution of these variables. We chose to use GLM instead of a linear regression model with log-transformed response because the interpretation of the parameters generated by the analysis are much easier to interpret than the geometric means generated by back transforming log linear regression parameters. In a GLM, the exponentiation of the mean response represents the ratio of the arithmetic means (AMR) of the compared categories which are in the same scale of the original response variable, *i.e.*, words or seconds [21].

First, we performed unadjusted analysis between coffee consumption and each cognitive function test (Model 1). Next the associations were adjusted for age, sex, and educational attainment (Model 2), and the associations that remained statistically significant in the Model 2 were additionally adjusted for alcohol consumption, cigarette smoking, diabetes status, hypertension status, coronary heart disease status, and LDL-cholesterol ratio (Model 3). The magnitudes of these associations were estimated by the difference of the mean or the AMR and their 95% confidence intervals (95%CI). Persons with missing values were excluded from these analyses. The analyses were conducted using the Stata 13.0 (Stata Corporation, College Station, TX, USA).

## 3. Results

The mean age of the study population was 51.96 (SD = 9.04) years and 54% were women. In total, 1398 persons (9.6%) never or almost never drank coffee, 4695 (32.2%) drank ≤1 cup/day, 5095 (35%) 2–3 cups/day, and 3375 (23.2%) ≥3 cups/day. Table 1 and Table 2 show the mean (or median) performance of participants in the learning, recall, and word recognition tests, semantic and phonemic verbal fluency tests and trail-making test B according to the amount of coffee intake. Even though learning, recall and word recognition tests and trail-making test B are statistically significant, there is no indication of variation in the mean (median) performance on these tests according to amount of coffee drank in adults and the elderly. Only the score on semantic and phonemic verbal fluency tests showed a dose-response relationship with the increase of the category of coffee consumption per day among elderly.

Table 3 shows the associations between coffee consumption and various cognitive function domains for the participants with 35–64 years old. In the univariable analysis (Model 1), only drinking ≤1 cups/day of coffee in the last 12 months was related to a reduction in the mean number of words pronounced in the semantic verbal fluency test (difference of the mean: −0.33; 95% CI: −0.66 to −0.00) and a 3% increase in the mean time taken to perform the trail-making test B (AMR: 1.03; 95% CI: 1.00 to 1.06) when comparing to never/almost never category. No other association was statistically significant in the univariable analysis. After adjustments for age, sex, and educational attainment (Model 2), drinking 2–3 cups/day of coffee was associated with a 1% increase in the mean number of words remembered on the learning, recall, and word recognition tests when compared to the never/almost never category (AMR: 1.01; 95%CI: 1.01 to 1.01). However, after full adjustments (Model 3), the statistical significance of the association between consuming 2–3 cups/day of coffee and the performance on the learning, recall, and word recognition tests became borderline (*p*-value = 0.052).

Table 4 shows the associations between coffee consumption and the performance on the cognitive function domains for the participants with 65–74 years old. In Model 1, coffee consumption equal to 2–3 cups/day was statistically related to 4% increase in the mean number of words remembered by participants in the learning, recall, and word recognition tests when compared to the never/almost never category (AMR: 1.04; 95%CI: 1.00 to 1.08). Also, those drinking ≥3 cups/day showed mean increase of almost two words (difference of the mean: 1.99; 95%CI: 0.81 to 3.18) in the mean number of words pronounced in the semantic and phonemic verbal fluency tests and mean increase of 1.36 words in the phonemic test (difference of the mean: 1.36; 95% CI: 0.29 to 2.43) when compared to the never/almost never category.

**Table 1:** Characteristics of the study population (35–64 years old) of ELSA-Brasil (2008–2010).

Variables	*Coffee Consumption (Cups/Day)*				
	Never or Almost Never	≤1 Cup/Day	2–3 Cups/Day	≥3 Cups/Day	*p*-Value
*Women, n (%)*	681 (52.9)	2263 (54.7)	2659 (59.3)	1531 (48.4)	**<0.001**
*Age* *(year)s, mean (SD)*	48.7 (7.6)	49.9 (7.6)	50.5 (7.5)	50.1 (6.9)	**<0.001**
*Educational Attainment, n (%)*					**<0.001**
Undergraduate school or more	686 (53.3)	2081 (50.3)	2429 (54.1)	1647 (52.0)	
Complete high school	469 (36.4)	1550 (37.5)	1595 (35.6)	1121 (35.4)	
Complete elementary school	88 (6.8)	279 (6.7)	250 (5.6)	215 (6.8)	
Incomplete elementary school	44 (3.4)	227 (5.5)	213 (4.8)	183 (5.8)	
*Alcohol Consumption, n (%)*					**<0.001**
Never or former user	542 (42.1)	1223 (29.6)	1305 (29.1)	821 (25.9)	
Moderate	674 (52.4)	2576 (62.3)	2895 (64.6)	2027 (64.0)	
Heavy	71 (5.5)	338 (8.2)	285 (6.34)	318 (10.0)	
*Smoking Status, n (%)*					**<0.001**
Never	913 (70.9)	2520 (60.9)	2749 (61.3)	1340 (42.3)	
Former	291 (22.6)	1208 (29.2)	1282 (28.6)	1015 (32.1)	
Current	83 (6.5)	409 (9.9)	456 (10.2)	811 (25.6)	
*Diabetes Status, n (%)*	234 (18.2)	752 (18.2)	764 (17.0)	561 (17.7)	0.529
*Hypertension Status, n (%)*	386 (30.0)	1489 (36.0)	1561 (34.8)	963 (30.4)	**<0.001**
*Coronary Heart Disease Status, n (%)*	19 (1.5)	83 (2.0)	81 (1.8)	56 (1.8)	0.638
*Low-density Lipoprotein-Cholesterol (mg/dl), mean (SD)*	128.20 (33.88)	131.27 (35.08)	131.73 (34.59)	132.90 (35.44)	0.198
*Learning, recall and word recognition tests (words), median (1° and 4° quartile)*	39 (34–42)	38 (34–42)	39 (35–42)	38 (34–42)	**0.027**
*Semantic verbal fluency test (words), mean (SD)*	18.87 (5.49)	18.54 (5.26)	18.62 (5.23)	18.72 (5.21)	**0.939**
*Phonemic verbal fluency test (words), mean (SD)*	12.62 (4.49)	12.61 (4.48)	12.61 (4.46)	12.69 (4.42)	0.878
*Trail-making test B (seconds), median (1° and 4° quartile)*	91 (70–127)	93 (72–132)	95 (72–129)	92 (71–129)	**0.0342**

* Performed Chi-square for frequencies, Analysis of variance for normal distribution and KrusKal-Wallis for non-normal distribution. Abbreviations: Longitudinal Study of Adult Health (ELSA-Brasil). Stardant Deviation:SD; analysis of variance: ANOVA.

**Table 2:** Characteristics of the study population (65–74 years old) of ELSA-Brasil (2008–2010).

Variables	*Coffee Consumption (Cups/Day)*				
	Never or Almost Never	≤1 Cup/Day	2–3 Cups/Day	≥3 Cups/Day	*p*-Value
*Women, n (%)*	56 (50.5)	305 (54.7)	305 (54.7)	317 (52.1)	**0.015**
*Age* *(years)*	69.34 (2.74)	68.81 (2.83)	68.7 (2.80)	68.22 (2.69)	**0.838**
*Educational Attainment, n (%)*					**0.012**
Under graduate school or more	60 (54.1)	296 (53.1)	341 (56.1)	144 (68.9)	
Complete high school	30 (27.0)	127 (22.7)	126 (20.7)	27 (12.9)	
Complete elementary school	9 (8.1)	64 (11.5)	69 (11.4)	14 (6.7)	
Incomplete elementary school	12 (10.8)	71 (12.7)	72 (11.8)	24 (11.5)	
*Alcohol Consumption, n (%)*					**<0.001**
Never or former user	59 (53.2)	186 (33.3)	210 (34.6)	53 (25.4)	
Moderate	46 (44.4)	340 (60.9)	362 (59.6)	139 (66.5)	
Heavy	6 (5.4)	32 (5.7)	35 (5.8)	17 (8.1)	
*Smoking Status, n (%)*					**<0.001**
Never	67 (60.4)	319 (57.3)	317 (52.1)	91 (43.5)	
Former	40 (36.0)	200(35.9)	247 (40.6)	82 (39.2)	
Current	4 (3.6)	38 (6.8)	44 (7.2)	36 (17.2)	
*Diabetes Status, n (%)*	42 (37.8)	194 (34.8)	210 (34.5)	73 (34.9)	0.928
*Hypertension Status, n (%)*	74 (66.7)	373 (66.9)	411 (67.6)	137 (65.6)	**0.959**
*Coronary Heart Disease Status, n (%)*	14 (12.6)	37 (6.6)	53 (8.7)	23 (11.0)	0.086
*Low-density LipoproteinCholesterol (mg/dl), mean (SD)*	125.27 (40.65)	128.23 (35.38)	125.74 (34.87)	131.22 (40.42)	**0.013**
*Learning, recall and word recognition tests (words), median (1° and 4° quartile)*	34.5 (29–38)	35 (30–40)	36 (32–40)	35 (30–39)	**0.048**
*Semantic verbal fluency test (words), mean (SD)*	16.14 (5.71)	16.55 (5.03)	17.00 (5.23)	18.14 (5.95)	**<0.001**
*Phonemic verbal fluency test (words), mean (SD)*	10.87 (4.85)	11.06 (4.56)	11.39 (4.71)	12.23 (4.40)	0.553
*Trail-making test B (seconds), median (1° and 4° quartile)*	110 (90–150)	120 (89–164)	114 (87.5–156)	101 (78–132)	**<0.001**

* Performed Chi-square for frequencies, Analysis of variance for normal distribution and KrusKal-Wallis for non normal distribution. Abbreviations: Longitudinal Study of Adult Health (ELSA-Brasil).

**Table 3:** Associations between coffee consumption and learning, recall, and word recognition tests, phonemic verbal fluency tests, and trail-making test B among the participants (35 to 64 years of age) of ELSA-Brasil (2008–2010).

	Coffee Consumption (Cups/Day)					
Cognitive Function Tests	Model 1		Modelo 2		Model 3	
	Coefficient (95%CI)	*p*-Value	Coefficient (95%CI)	*p*-Value	Coefficient (95%CI)	*p*-Value
***Learning, Recall, and word recognition tests (n = 12,957) ^a^***						
Never/almost never	Reference		Reference		Reference	
≤1 cup/day	0.99 (0.99 1.01)	0.990	1.00 (0.99 1.01)	0.094	1.00 (0.99 1.01)	0.175
2–3 cups/day	1.00 (0.99, 1.02)	0.113	1.01 (1.00, 1.01)	**0.025**	1.00 (0.99, 1.01)	0.052
≥3 cups/day	0.99 (0.99, 1.02)	0.404	1.00 (0.99, 1.01)	0.192	1.00 (0.99, 1.01)	0.073
***Semantic verbal fluency test (n = 13,037) ^b^***					-	-
Never/almost never	Reference		Reference			
≤1 cup/day	−0.33 (−0.66, −0.00)	**0.049**	−0.04 (−0.33, 0.25)	0.773		
2–3 cups/day	−0.25 (−0.58, 0.70)	0.124	−0.10 (−0.39, 0.18)	0.483		
≥3 cups/day	−0.15 (−0.49, 0.18)	0.374	0.10 (−0.19, 0.41)	0.497		
***Phonemic verbal fluency test (n = 13,018) ^b^***					-	-
Never/almost never	Reference		Reference			
≤1 cup/day	−0.01 (−0.29, 0.26)	0.940	0.19 (−0.06, 0.44)	0.143		
2–3 cups/day	−0.00 (−0.28, 0.27)	0.974	0.08 (−0.17, 0.33)	0.525		
≥3 cups/day	0.06 (−0.22, 0.35)	0.647	0.25 (−0.00, 0.52)	0.056		
***Trail-making test B (n = 11,949) ^a^***					-	-
Never/almost never	Reference		Reference			
≤1 cup/day	1.03 (1.00, 1.06)	**0.025**	1.00 (0.97, 1.02)	0.935		
2–3 cups/day	1.02 (0.99, 1.05)	0.109	1.00 (0.97, 1.02)	0.736		
≥3 cups/day	1.01 (0.98, 1.03)	0.285	0.98 (0.95, 1.00)	0.105		

**Model 1:** Unadjusted model **Model 2**: Adjusted for age, sex and educational attainment. **Model 3**: Model 2 + hypertension status, coronary heart disease status, diabetes status, smoking status, alcohol consumption and Low-density Lipoprotein cholesterol. ^a^ Performed generalized linear model (arithmetic mean ratio); ^b^ Performed linear regression (difference of the mean). Abbreviations: Longitudinal Study of Adult Health (ELSA-Brasil); Confidence Interval (CI).

**Table 4:** Associations between coffee consumption and learning, recall, and word recognition tests, phonemic verbal fluency tests, and trail-making test B among the participants (65 to 74 years of age) of ELSA-Brasil (2008–2010).

	Coffee Consumption (Cups/Day)					
Cognitive Function Tests	Model 1		Model 2		Model 3	
	Coefficient (95%CI)	*p*-Value	Coefficient (95%CI)	*p*-Value	Coefficient (95%CI)	*p*-Value
***Learning, Recall, and word recognition tests (n = 1458) ^a^***						
Never/almost never	Reference		Reference		Reference	
≤1 cup/day	1.02 (0.98 1.06)	0.320	1.01 (0.98 1.05)	0.290	1.01 (0.98 1.05)	0.335
2–3 cups/day	1.04 (1.00, 1.08)	**0.031**	1.04 (1.00, 1.07)	**0.026**	1.03 (1.00, 1.07)	**0.034**
≥3 cups/day	1.02 (0.97, 1.06)	0.353	1.00 (0.96, 1.04)	0.731	1.01 (0.97, 1.05)	0.569
***Semantic verbal fluency test (n = 1481) ^b^***						
Never/almost never	Reference		Reference		Reference	
≤1 cup/day	0.40 (−0.64, 1.46)	0.445	0.44 (−0.47, 1.37)	0.343	0.34 (−0.59, 1.27)	0.473
2–3 cups/day	0.85 (−0.19, 1.89)	0.109	0.73 (−0.18, 1.65)	0.115	0.65 (−0.27, 1.58)	0.165
≥3 cups/day	1.99 (0.81, 3.18)	**0.001**	1.29 (0.24, 2.35)	**0.016**	1.23 (0.16, 2.29)	**0.023**
***Phonemic verbal fluency test (n = 1477) ^b^***					-	-
Never/almost never	Reference		Reference			
≤1 cup/day	0.18 (−0.75, 1.13)	0.697	0.29 (−0.53, 1.12)	0.487		
2–3 cups/day	0.51 (−0.42, 1.45)	0.279	0.48 (−0.33, 1.30)	0.247		
≥3 cups/day	1.36 (0.29, 2.43)	**0.012**	0.85 (−0.08, 1.79)	0.076		
***Trail-making test B (n = 1172) ^a^***					-	-
Never/almost never	Reference.		Reference.			
≤1 cup/day	1.08 (0.98, 1.19)	0.104	1.07 (0.99, 1.16)	0.084		
2–3 cups/day	1.05 (0.96, 1.16)	0.246	1.06 (0.98, 1.15)	0.134		
≥3 cups/day	0.93 (0.83, 1.04)	0.209	0.99 (0.90, 1.06)	0.901		

**Model 1:** Unadjusted model **Model 2:** Adjusted for age, sex and educational attainment. **Model 3:** Model 2 + hypertension status, coronary heart disease status, diabetes status, smoking status, alcohol consumption and Low-density Lipoprotein cholesterol. ^a^ Performed generalized linear model (arithmetic mean ratio); ^b^ Performed linear regression (difference of the mean). Abbreviations: Longitudinal Study of Adult Health (ELSA-Brasil); Confidence Interval (CI).

In Model 2 and also in Model 3, after full adjustments, coffee consumption remained associated with 4% and 3% increases, respectively, in the mean number of words remembered on learning, recall, and word recognition tests when compared to the 2–3 cups/day to never/almost never category (AMR in Model 2: 1.04; 95% CI: 1.00 to 1.07 and AMR in Model 3: 1.03; 95% CI: 1.00 to 1.07). Drinking ≥3 cups/day of coffee remained associated with an increase in the mean number of words pronounced in semantic verbal fluency test when comparing to the never/almost never category (difference of the man: 1.29; 95% CI: 0.24 to 2.35 (Model 2) and difference of the mean: 1.23; 95% CI: 0.16 to 2.29 (Model 3)) (Table 4). Drinking coffee was neither associated the duration of performing trail-making B test nor to the number of words pronounced in the phonemic test.

## 4. Discussion

We examined the relations between the amount of coffee consumption in the last 12 months and performance on six cognitive tests spanning the domains of verbal memory, efficiency of searching in long-term memory, language and executive function in adults and older participants of a cohort of free living Brazilians. After full adjustments we found that among elderly, drinking 2–3 cups of coffee per day was associated with about a 3% increase in the mean number of words remembered on the learning, recall and word recognition tests. Also, that drinking ≥3 cups/day of coffee was associated with an increase of about 1.23 words in the mean number of words pronounced in the semantic verbal fluency test. However, there was no indication of a dose response relationship in these associations.

Coffee is one of the most commonly consumed beverages by Brazilians and a major source of antioxidants and stimulants in the diet. According to a recent survey, the estimated average usual daily coffee intake from the total population was 163 (Standart Erro (SE) = 2.8) mL, and the median was 129 mL, with the 5th and 95th percentiles 3 mL (SE = 0.5) and 442 (SE = 7.9) mL, respectively [3]. The average intake in the ELSA-Brasil cohort (149.35 mL, SE = 1.1) is thus close to that of the overall population in the country.

Besides caffeine, coffee contains many other substances, like magnesium and many phenolic acids, chlorogenic acid being the most abundant [22]. Consumption of coffee increases the antioxidant capacity in plasma [23,24], which may provide a protective effect against free radicals that cause oxidative damage to neurons, which appear to be very vulnerable to the effects of free radicals [25]. A previous study of ELSA-Brasil found a protective effect of coffee consumption on the risk of adult-onset diabetes [26], and diabetes is associated with the occurrence of vascular dementia [27]. Cao and colleagues (2012) [2] observed in a case-control study that caffeine/coffee intake is associated with a reduced risk of dementia or delayed onset, particularly for those who already have mild cognitive impairment.

In this cross sectional analysis we observed that coffee consumption was not associated with adult cognition. Among elderly, it was related to the verbal memory and efficiency of searching in long-term memory, but the associations are significant for only one category of exposure and they are not the same for each cognitive test. A cross-sectional study among older adults in Singapore found no association between coffee consumption and the performance on any of the four specific domains of cognitive function that they examined [28]. Longitudinal studies with older adults that evaluated the relationship between coffee consumption and cognitive decline also found inconsistent results, including no relationship [29,30], or isolated statistically significant findings without dose-response gradients [31,32]. A systematic review of cross sectional and cohort studies showed a protective effect of coffee, tea, and caffeine consumption against late-life cognitive impairment/decline in some cognitive domains but without a distinct dose-response association [33]. A meta-analysis of three longitudinal studies considering different measures of coffee intake conducted by Santos and colleagues (2010) [34] showed no association between the amount of coffee drank and cognitive decline. However, a study with elderly reported that frequent coffee drinking was associated with a small attenuation in the rate of cognitive decline in women [35].

Nonetheless, it is important to consider that the lack of a dose response relationship or of consistency in the results of ours and other studies on the association of coffee intake and cognition might express the effect of other compounds also present in coffee, such as the lipid fraction (cafestol, kahweol and especially 16-O-methylcafestol) which can have detrimental effects and thus mask linear association with the amount of coffee intake [36]. In fact, from a practical point of view, the question of what the overall effect of coffee is on cognition especially in each period of life, and what the specific domains potentially affected by this beverage are, remains unmeasured.

Likewise other studies on dietary factors, the present study has limitations, as measurement errors are inevitable. Individuals poorly remember their usual consumption of foods and beverages if memory is affected by regular coffee consumption, than misclassification may more common among those with lower performance on this domain, contributing to bias any potential association towards the null. This concern is in addition to the potential effect of unmeasured confounders, such as others beverages and foods which contain caffeine and residual confounding that is inevitable in studies of this nature. However, the advantage of large epidemiological studies data that take into account multiple biological, environmental, and clinical confounding factors is to allow adjustment for confounding factors that can obscure the true cause of this an association. The prevalence of coffee drinking in the present study is likely to differ from that found in the Brazilian population as a whole, because the ELSA-Brasil cohort was not intended to be representative of the country population. For instance, the cohort does not include the unemployed and has a much higher percentage of people with university degrees. As extensively debated recently, sampling representativeness is necessary when the study aims to estimate the prevalence of a condition in a given population, which is not the objective of the present study, but it is not required to draw valid scientific inferences from exposure-outcome associations found in well conducted epidemiological studies [37,38].

We reported that the association of coffee intake with better verbal memory and efficiency of searching in long-term memory persisted in the elderly even when all known potential confounding factors (age, education, gender, diabetes, hypertension, LDL-Cholesterol, cardiovascular disease, smoking, and alcohol use) were taken into account. The major strengths of this study are its sample size and the opportunity to adjust for several possible confounding factors and to analyze if the potential effect of coffee on cognition is only present among the elderly. The ELSA-Brasil is a longitudinal study with a wide battery of cognitive function tests and will allow to evaluate whether coffee consumption either delays or reduces the speed of cognitive decline in different domains of cognition with time.

## 5. Conclusions

Our results suggest that coffee consumption is associated with better cognitive performance on memory and efficiency of searching in long-term memory only in elderly, but without a dose response relationship. Before advocating the benefits of coffee on verbal memory further research is needed, especially prospective studies and studies in brain imaging in humans, to fully understand the nature of these associations and rule out confounding by other factors.
